# Differentially expressed mRNAs, lncRNAs, and miRNAs with associated co-expression and ceRNA networks in ankylosing spondylitis

**DOI:** 10.18632/oncotarget.22708

**Published:** 2017-11-27

**Authors:** Chen Zhang, Chen Wang, Zhenyu Jia, Wenwen Tong, Delin Liu, Chongru He, Xuan Huang, Weidong Xu

**Affiliations:** ^1^ Department of Orthopedics, Changhai Hospital, Second Military Medical University, Shanghai, China

**Keywords:** ankylosing spondylitis, microarray, competing endogenous RNA, long noncoding RNA, miRNA

## Abstract

Ankylosing spondylitis (AS) is a chronic autoimmune disease characterized by systemic inflammation and pathological osteogenesis. However, the genetic etiology of AS remains largely unknown. This study aimed to explore the potential role of coding and noncoding genes in the genetic mechanism of AS. Using microarray analyses, this study comprehensively compared lncRNA, microRNA, and mRNA profiles in hip joint ligament tissues from patients with AS and controls. A total of 661 lncRNAs, 574 mRNAs, and 22 microRNAs were differentially expressed in patients with AS compared with controls. Twenty-two of these genes were then validated using real-time polymerase chain reaction. Gene ontology and pathway analyses were performed to explore the principal functions of differentially expressed genes. The pathways were involved mainly in immune regulation, intercellular signaling, osteogenic differentiation, protein synthesis, and degradation. Gene signal transduction network, coding–noncoding co-expression network, and competing endogenous RNA expression network were constructed using bioinformatics methods. Then, two *miRNAs*, *miR-17-5p* and *miR-27b-3p*, that could increase the osteogenic differentiation potentials of ligament fibroblasts were identified. Finally, differentially expressed, five lncRNAs, four miRNAs, and five mRNAs were validated using quantitative real-time polymerase chain reaction. These results suggested that mRNAs, lncRNAs, and microRNAs were involved in AS pathogenesis. The findings might help characterize the pathogenesis of AS and provide novel therapeutic targets for patients with AS in the future.

## INTRODUCTION

Ankylosing spondylitis (AS) is a chronic autoimmune disease characterized by systemic inflammation and pathological osteogenesis [1]. It initially involves the sacroiliac joints, and affects mainly the axial skeleton, tendon attachment points, and ligaments [2]. AS eventually leads to ligamentous ossifications and spinal fusion, which bring a great burden to patients and society. Several studies have explored the pathogenesis of inflammation in AS. It has been demonstrated to involve bacterial infection [3], macrophage activation [4], certain cytokines [5–9], HLA-B27 misfolding [10], and autophagy [11, 12]. However, the real cause of the disease is still unknown. Also, the mechanism of new bone formation remains poorly understood. Therefore, in-depth studies on the precise mechanisms of inflammation and new bone formation in AS should provide valuable information.

Ligaments are band of tissues that hold the ends of bones together at a joint, allowing most joints to move and stabilizing them. They are hypothesized to be the primary target tissue for inflammation and ossification in AS, and are responsible for many of the symptoms in patients with AS [13, 14]. A previous study has identified alterations in the ligaments of patients with AS, including calcification of tissue, significant increases in microvascular density, and irregular arrangements of collagen fibrils [15]. Fibroblasts are the most common cells of ligaments around joints. Recently, some studies demonstrated that fibroblasts contributed to pathological osteogenesis, partly because they expressed osteogenesis-associated genes [16] and had the osteogenic potential to be induced to osteoblasts [17]. Recently, the osteogenic potential of fibroblasts was demonstrated to be greater in patients with AS than in patients with osteoarthritis (OA) [18]. However, the specific molecular changes in ligaments from patients with AS remain to be elucidated.

Over the past decades, noncoding RNAs (ncRNAs), including long ncRNAs (lncRNAs) and short ncRNAs, have gained attention. MicroRNAs (miRNAs) are small noncoding RNAs (∼22 nucleotides long) that mostly downregulate the expression of target genes at the posttranscriptional level [19]. MiR-10b-5p was found to be a novel T helper (Th) 17 regulator present in Th17 cells from AS [20]. In addition, serum miR-146a, miR-29a, and miR-155 were significantly upregulated in patients with AS [21, 22]. Dysregulation of these miRNAs in the circulation could be potential biomarkers for AS. However, no studies have focused on miRNA profiles of diseased tissues. LncRNAs are a class of noncoding RNAs more than 200 nucleotides long [23]. Many studies have demonstrated the involvement of lncRNAs in inflammation and osteogenesis. Altered expression of lncRNAs has been implicated in the pathogenesis of different forms of arthritis, including rheumatoid arthritis (RA) [24] and OA [25]. However, the particular functions of lncRNAs in the pathogenesis of AS remain unclear. Recently, the competing endogenous RNA (ceRNA) hypothesis was proposed according to which lncRNAs and other RNA molecules harboring miRNA response elements could compete with one another for binding to a common miRNA, thus regulating miRNA-mediated gene silencing [26]. The involvement of ceRNA network has been demonstrated in several diseases [27–31], but it has not yet been explored in AS.

In the present study, a microarray analysis was performed to compare the hip joint ligament tissues of patients with AS (the AS group) and patients with femoral neck fracture (the control group), and to identify differentially expressed (DE) lncRNAs, miRNAs, and mRNAs. Gene Ontology (GO) categories and Kyoto Encyclopedia of Genes and Genomes (KEGG) pathway enrichment analyses were used to explore specific functions of lncRNAs, miRNAs, and mRNAs. A gene–gene interaction network and lncRNA–mRNA co-expression networks were constructed. The ceRNA regulation network in AS was systematically investigated through co-expression and miRNA-binding prediction analyses. Finally, two DE miRNAs were found to be involved in osteogenic differentiation of ligament fibroblasts. The findings offered greater insights into the pathogenesis of AS and potentially provided new therapeutic targets.

## RESULTS

### DE lncRNAs, mRNAs, and miRNAs

**Figure 1:**
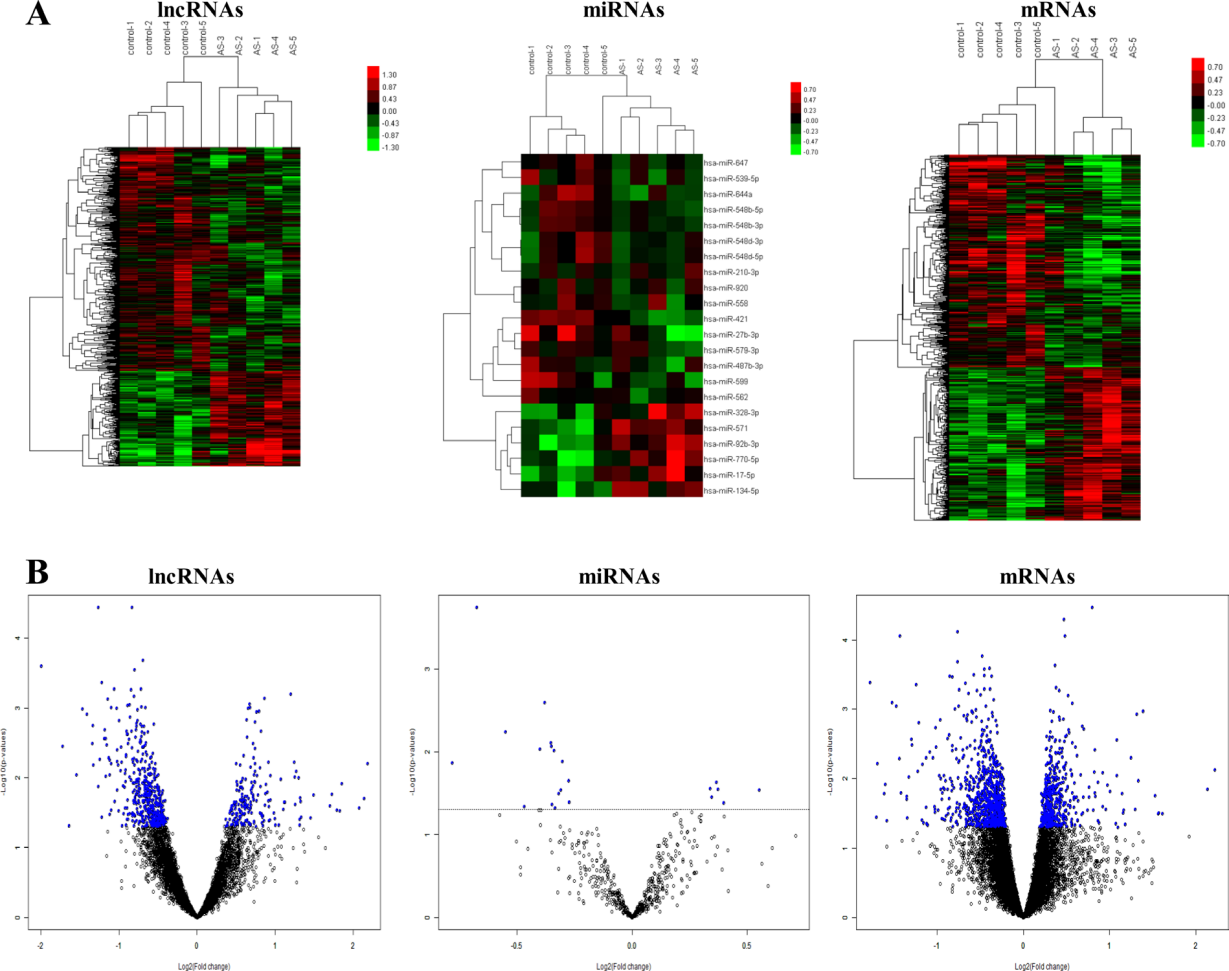
Heatmap and volcano plots showing lncRNA, mRNA, and miRNA levels (**A**) Screening criteria were as follows: *P* ≤ 0.05 for lncRNAs, miRNAs, and mRNAs. Expression values are depicted in line with the color scale; intensity increases from green to red. Each column represents one sample, and each row indicates a transcript. (**B**) Volcano plots reflecting number, significance, and reliability of differentially expressed lncRNAs, miRNAs, and mRNAs. The abscissa is log2 (FC value) and the ordinate is –log10 (*P* value). Blue dots are deregulated genes, and black dots are genes that were the same between the two groups.

A total of 661 lncRNAs, 574 mRNAs, and 22 miRNAs were found to be DE in the AS group compared with the control group. Of these, 240 mRNAs, 6 miRNAs, and 196 lncRNAs were overexpressed and 334 mRNAs, 16 miRNAs, and 465 lncRNAs were downexpressed in patients with AS compared with controls. The hierarchical clustering analysis of these RNAs is presented in Figure 1A, which shows that the three kinds of RNAs could well distinguish patients with AS from the controls. Finally, volcano plots were constructed to demonstrate differences between the two groups (Figure 1B).

### GO and KEGG enrichment analysis of DE miRNA target genes and DE mRNAs

**Figure 2:**
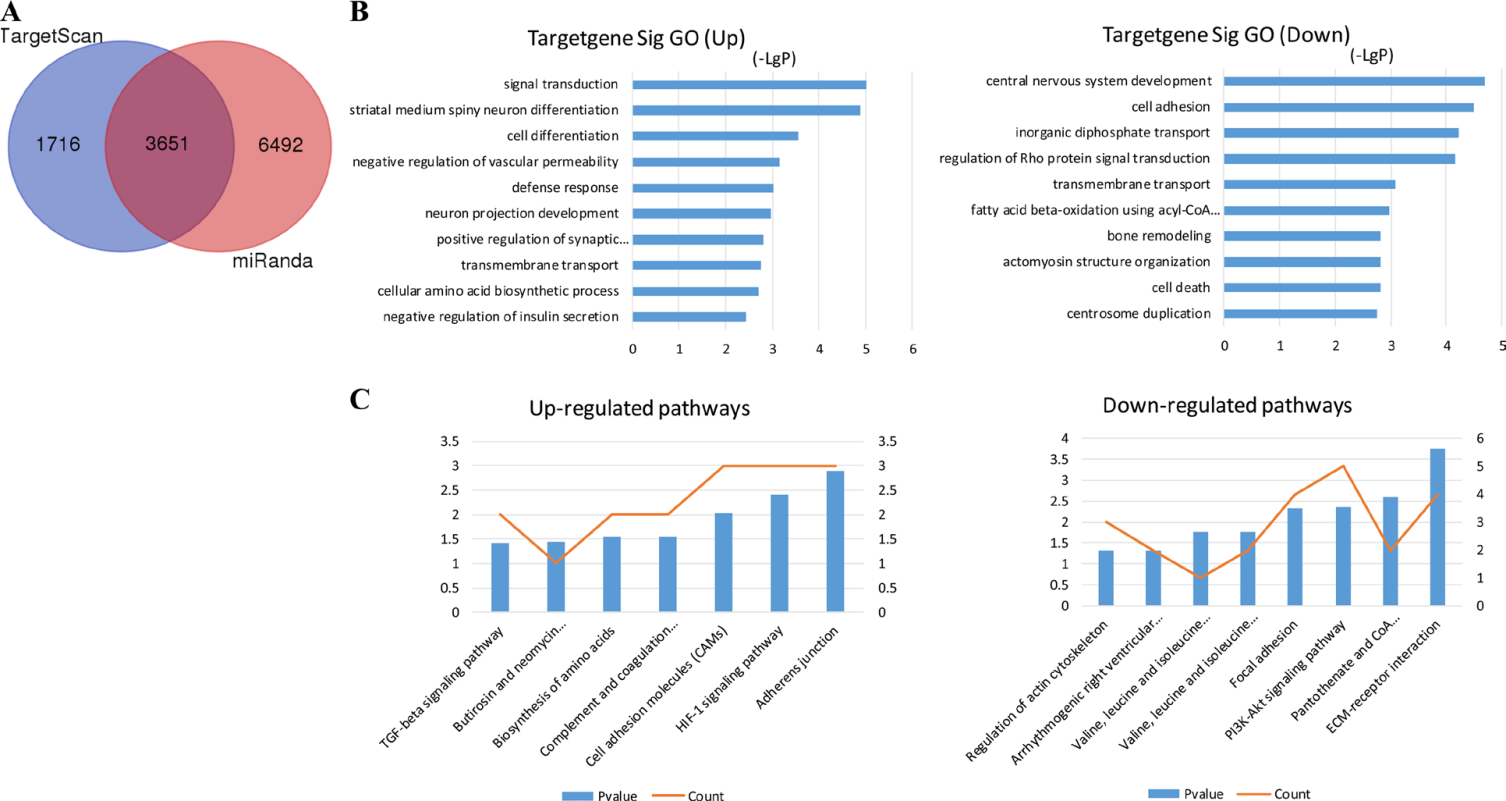
MiRNA target prediction and enrichment analysis of the predicted target genes (**A**) Venn diagram of genes targeted by differentially expressed miRNAs. TargetScan and miRanda databases were used to predict miRNA targets. The numbers of predicted target genes were presented in a Venn diagram. (**B**) GO annotation of upregulated and downregulated mRNAs, with the top 10 functional GO terms. (**C**) Significant pathways of upregulated and downregulated mRNAs. Enrichment score values were calculated as –log10 (*P* values). GO, Gene Ontology.

**Figure 3:**
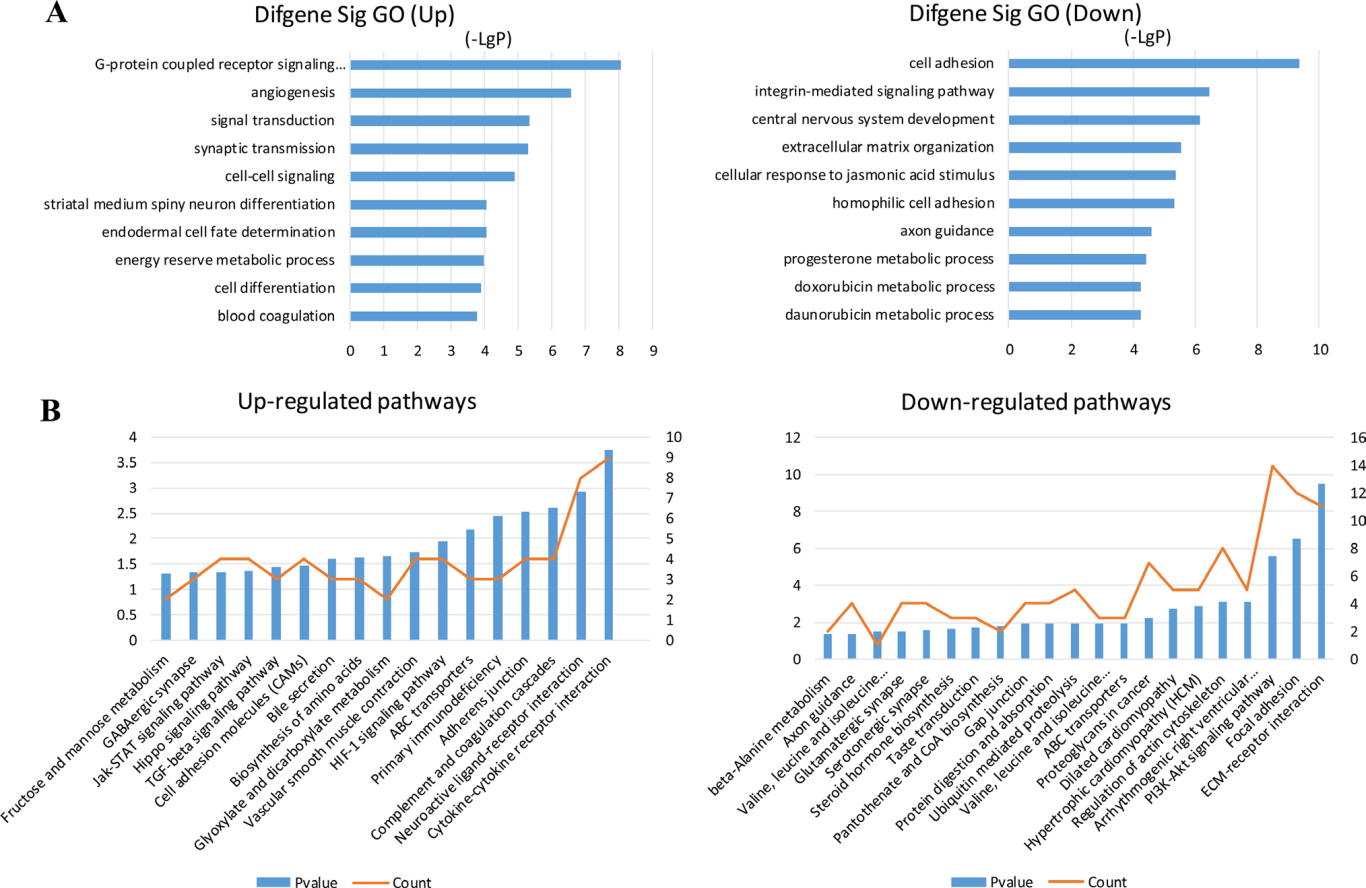
GO and KEGG pathway analysis of the differentially expressed mRNAs (**A**) GO annotation of upregulated and downregulated mRNAs, with the top 10 functional GO terms. (**B**) Significant pathways of upregulated and downregulated mRNAs. Enrichment score values were calculated as –log10 (*P* values). GO, Gene Ontology; KEGG, Kyoto Encyclopedia of Genes and Genomes.

Target prediction of DE miRNAs was carried out using two online software programs, miRanda and TargetScan. The results of the two target prediction programs were integrated, and only the intersection was selected to increase the specificity. As a result, 3651 target genes were predicted for 16 miRNAs (Figure 2A). Then, GO analyses were performed on the DE miRNA target genes and DE mRNAs. The upregulated and downregulated genes were individually analyzed. The results showed that 166 and 123 GO functions were annotated for upregulated and downregulated miRNA targets. The top 10 statistically significant GO terms are shown in Figure 2B. The results showed that 255 and 247 GO functions were annotated for upregulated and downregulated mRNAs. The top 10 statistically significant GO terms are shown in Figure 3A. Next, the DE miRNA target genes and DE mRNAs were assessed with the KEGG database. The pathways are shown in Figures 2C and 3B (DE miRNA target genes and DE mRNAs, respectively). The predicted target genes were involved in adherens junction, hypoxia-inducible factor-1 (HIF-1) signaling pathway, transforming growth factor-β (TGF-β) signaling pathway, extracellular matrix–receptor interaction, focal adhesion, and so forth. In addition, the DE mRNAs were also enriched in biological pathways, such as cytokine–cytokine receptor interaction, TGF-β signaling pathway, and Hippo signaling pathway.

### Identification of miRNA–gene network and miRNA–GO network

**Figure 4:**
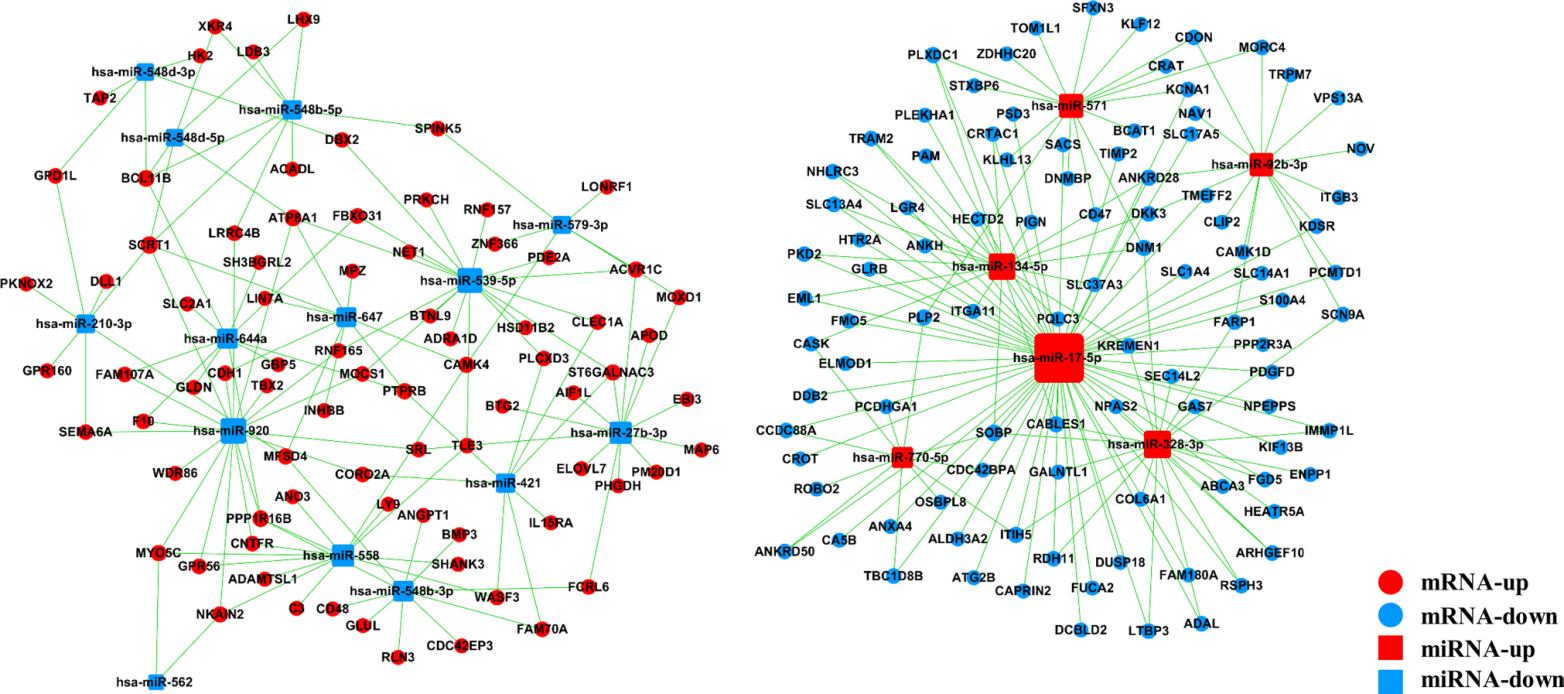
MicroRNA–gene network *Box* nodes denote miRNAs (*red*: upregulated miRNAs; *blue*: downregulated miRNAs), *circle* nodes denote genes (*red*: upregulated genes; *purple*: downregulated genes), and *edges* show the inhibitory effect of miRNAs on genes. Degree means the contribution of one miRNA to the genes around or the contribution of one gene to the miRNAs around. The key miRNAs and genes in the network always have the highest degrees.

**Figure 5:**
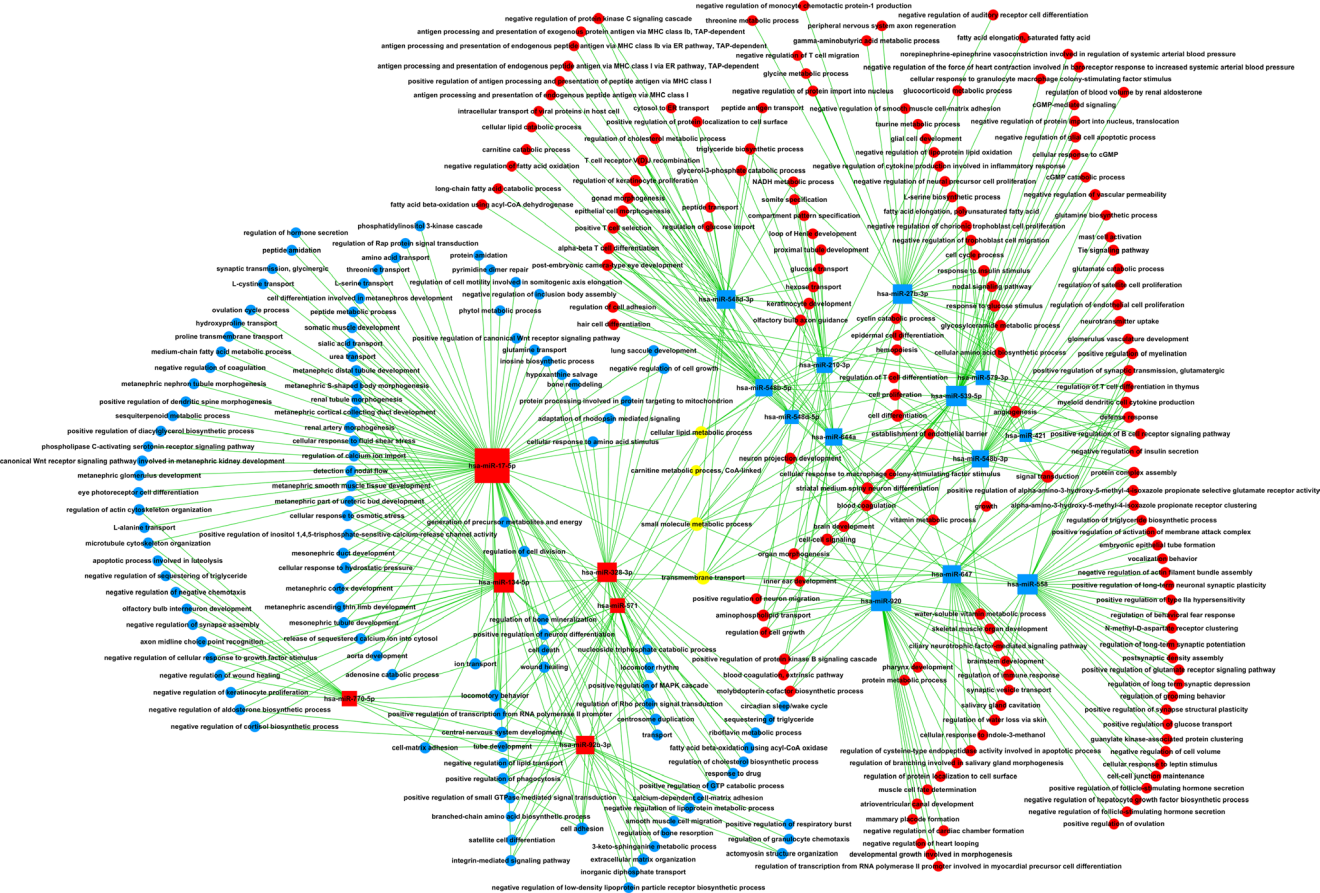
MicroRNA–GO network *Box* nodes denote miRNAs (*red*: upregulated miRNAs; *blue*: downregulated miRNAs), *circle* nodes denote GOs (*red*: upregulated GOs; *purple*: downregulated GOs), and *edges* show the inhibitory effect of miRNAs on GOs. Degree means the contribution of one miRNA to the GOs around. The key miRNAs in the network always have the highest degrees. GO, Gene Ontology.

The miRNA–gene network was built to show the interactions of miRNAs and their target genes. The genes were identified from the intersection between 2095 putative target genes of these miRNAs and 574 DE mRNAs. As shown in Figure 4, *miR-17-5p* was found to be more prominent and crucial in the network because its targets were more than 50. Furthermore, the miRNA–GO network was constructed according to the relationships among significant GOs and miRNAs (Figure 5). *MiR-17-5p* seemed to be the key node in this network, which regulated 87 biological processes.

### Signal network and coding–noncoding gene co-expression network

**Figure 6:**
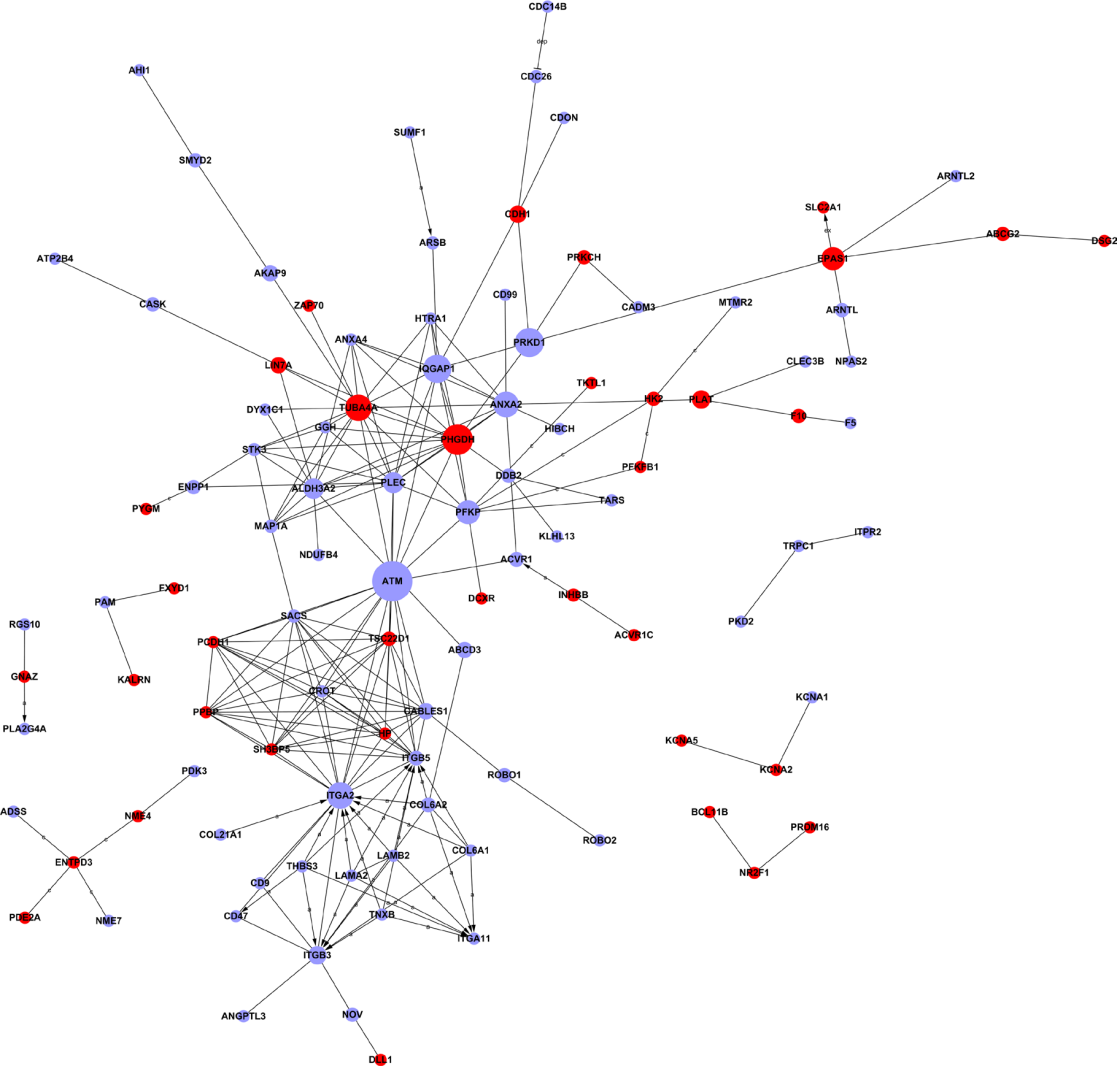
Interaction network of differentially expressed genes (signal-net) In the signal-net, the genes are characterized by measuring their “betweenness centrality”: the number of times a node is located in the shortest path between two other nodes. This measure reflects the importance of a node in a network in relation to another. The *circles* represent important functional genes (*red*: upregulated genes; *purple*: downregulated genes); the circle size represents the degree of interaction (betweenness centrality), and lines indicate the interactions.

The gene signal transduction network was established based on the significant GO and pathway analysis to investigate the relationship of DE genes and their potential role in AS (Figure 6). The genes with the highest betweenness centralities (≥0.01) are listed in Table 1. *ATM* (downregulated), *PHGDH* (upregulated), *ITGA2* (downregulated), and *TUBA4A* (upregulated) were the four key genes identified by the analysis. *ITGA2* had the highest degree of connectivity with other genes. However, *ATM* and *PHGDH* were the key nodes and had the highest betweenness centralities in the signal-net. Finally, the coding–noncoding gene co-expression (CNC) network was constructed to identify the interactions between lncRNAs and mRNAs. A total of 521 lncRNAs and 328 mRNAs were used to build the CNC network for the AS group (Supplementary Figure 2) and 547 lncRNAs and 323 mRNAs to build the network for the control group (Supplementary Figure 3). According to the Diffk values (|Diffk| ≥ 0.75) for the 2 networks, 24 genes, including 17 lncRNAs and 7 mRNAs, showed different connectivities between the AS and control groups, indicating that their roles were important in the core pathways of AS (Table 2).

**Table 1:** Top genes ranked by betweenness centrality over 0.1 after analysis of signal-net

Gene symbol	Description	Betweenness centrality	Degree	style
ATM	Ataxia telangiectasia mutated	0.0921211	16	Down
PHGDH	Phosphoglycerate dehydrogenase	0.0606976	15	Up
PRKD1	Protein kinase D1	0.0550501	5	Down
IQGAP1	IQ motif containing GTPase activating protein 1	0.0530205	11	Down
ITGA2	Integrin, alpha 2 (CD49B, alpha 2 subunit of VLA-2 receptor)	0.0473438	20	Down
TUBA4A	Tubulin, alpha 4a	0.0472054	16	Up
ANXA2	Annexin A2	0.0441149	11	Down
PFKP	Phosphofructokinase, platelet	0.0385101	10	Down
EPAS1	Endothelial PAS domain protein 1	0.0365287	5	Up
PLEC	Plectin	0.0286934	14	Down
ALDH3A2	Aldehyde dehydrogenase 3 family, member A2	0.027284	11	Down
ITGB3	Integrin, beta 3 (platelet glycoprotein IIIa, antigen CD61)	0.0205254	11	Down
PLAT	Plasminogen activator, tissue	0.0201645	3	Up
CDH1	Cadherin 1, type 1, E-cadherin (epithelial)	0.0167665	4	Up
CABLES1	Cdk5 and Abl enzyme substrate 1	0.0135026	11	Down
LIN7A	Lin-7 homolog A (C. elegans)	0.0135026	4	Up
AKAP9	A kinase (PRKA) anchor protein (yotiao) 9	0.0135026	2	Down
ABCD3	ATP-binding cassette, subfamily D (ALD), member 3	0.0124869	2	Down

**Table 2:** Twenty-four genes with different pathway connectivities (identified with Diffk values) in patients with AS and controls (|Diffk| ≥ 0.75)

Gene symbol	Description	Style	|Diffk|	Type
SMYD5-AS2	---	down	1	noncoding
SLC2A1	solute carrier family 2 (facilitated glucose transporter), member 1	up	0.875	coding
PDE4D	processed_transcript	down	0.875	noncoding
SLC9A9	solute carrier family 9, subfamily A (NHE9, cation proton antiporter 9), member 9	down	0.875	coding
CSNK1D-AS8	---	up	0.8125	noncoding
SLC35F5	solute carrier family 35, member F5	down	0.8125	coding
BRD8-AS1	---	down	0.8125	noncoding
PCID2-AS7	---	down	0.8125	noncoding
REV1-AS3	---	down	0.8125	noncoding
OPN1SW-AS1	---	down	0.8125	noncoding
CHD1L-AS7	---	down	0.8125	noncoding
TARBP1-AS7	---	down	0.8125	noncoding
VDAC2	retained_intron	down	0.8125	noncoding
REXO2-AS1	---	down	0.75	noncoding
NDRG1-AS6	---	up	0.75	noncoding
MFF-AS2	---	down	0.75	noncoding
WASHC4	nonsense_mediated_decay	down	0.75	noncoding
SPINK5	serine peptidase inhibitor, Kazal type 5	up	0.75	coding
HSD3B7	hydroxy-delta-5-steroid dehydrogenase, 3 beta- and steroid delta-isomerase 7	down	0.75	coding
PRDM16	PR domain containing 16	up	0.75	coding
CD46-AS9	---	down	0.75	noncoding
RPLP0-AS3	---	up	0.75	noncoding
MGAT5-AS2	---	down	0.75	noncoding
VASH1	vasohibin 1	down	0.75	coding

### Construction of ceRNA network

**Figure 7:**
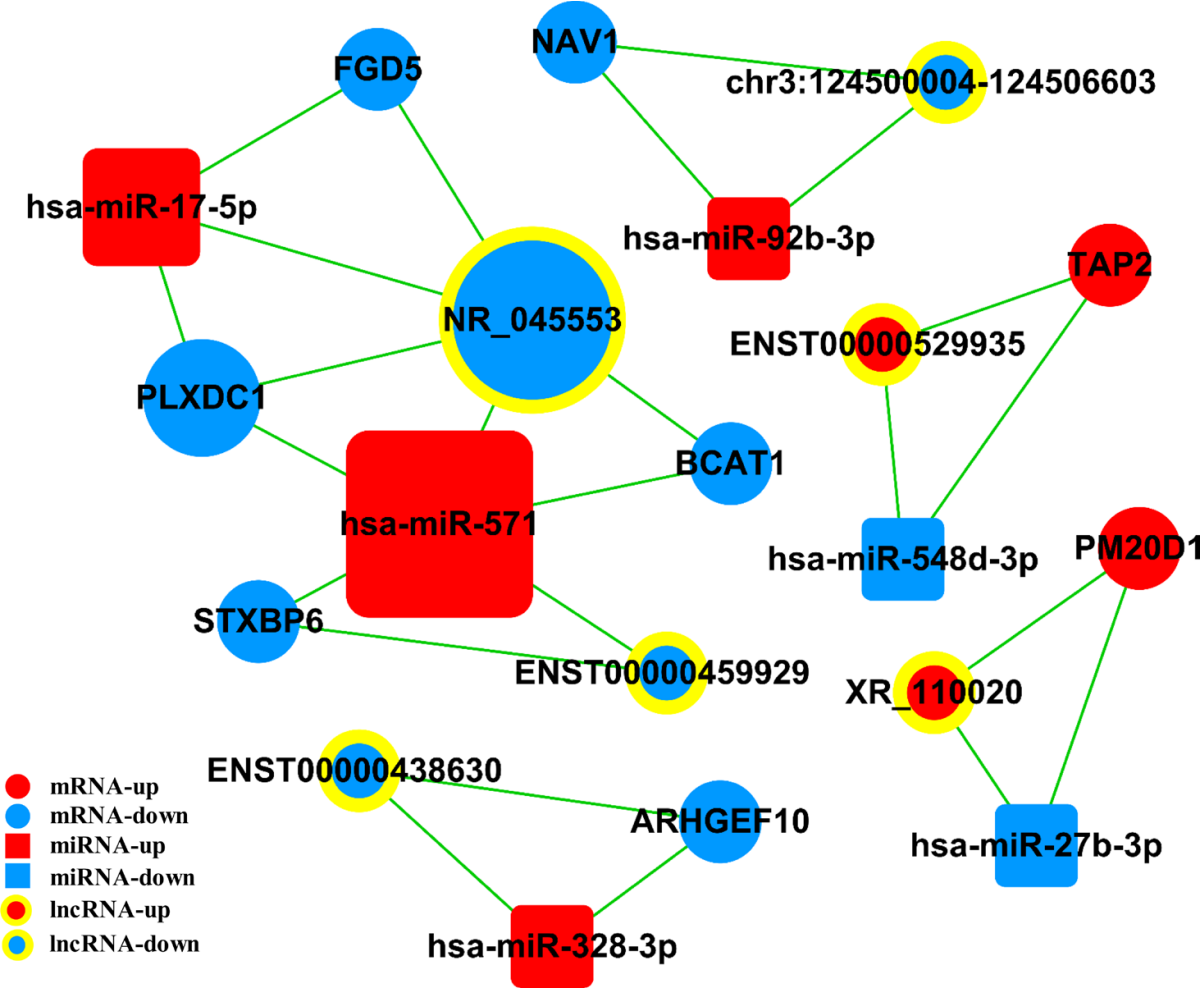
Competing endogenous RNA network in AS The competing endogenous RNA network is based on miRNA–mRNA, miRNA–lncRNA, and lncRNA–mRNA interactions. Boxes represent miRNAs, circles represent mRNAs, and concentric circles represent lncRNAs. Red represents upregulated and blue represents downregulated genes. AS, Ankylosing spondylitis.

The ceRNA network was constructed based on co-expressed lncRNAs–miRNAs, miRNAs–mRNAs, and lncRNAs–mRNAs. As shown in Figure 7, the lncRNA–miRNA–mRNA network was composed of six lncRNAs, eight mRNAs, and six miRNAs.

### Validation of DE lncRNAs, mRNAs, and miRNAs

**Figure 8:**
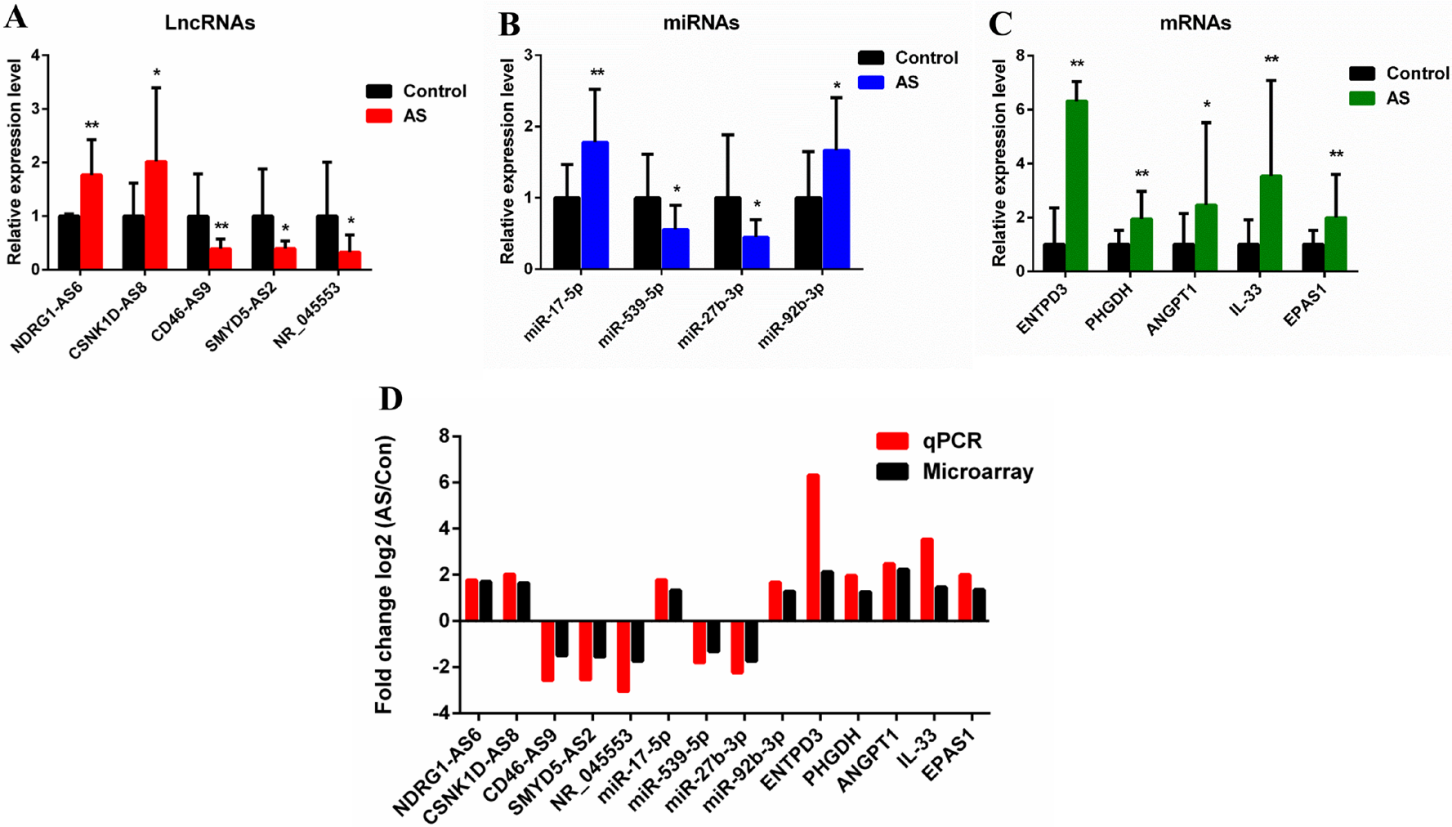
Validation of microarray data by qRT-PCR (**A**) Five lncRNAs, (**B**) four miRNAs, and (**C**) five mRNAs were validated by qRT-PCR between AS and control groups. The relative expression level of each RNA was normalized. The data displayed in histograms are expressed as means ± standard deviation. ^*^P < 0.05; ^**^P < 0.01 compared with the control group. (**D**) Comparison between qRT-PCR results and microarray data revealing a good correlation between the two methods. The heights of the columns represent the fold changes (log2 transformed) computed using the qRT-PCR and microarray data. qRT-PCR, Quantitative real-time polymerase chain reaction.

Based on the microarray data, five lncRNAs, four miRNAs, and five mRNAs were randomly selected for quantitative real-time polymerase chain reaction (qRT-PCR) verification. DE mRNAs were identified in ligament tissue samples from 15 patients with AS and 24 control subjects. LncRNAs and miRNAs were identified in 15 pairs of tissue samples. LncRNAs NDRG1-AS6 and CSNK1D-AS8 were upregulated, while CD46-AS9, SMYD5-AS2, and NR_045553 were downregulated (Figure 8A). *MiR-17-5p* and *miR-92b-3p* were upregulated, while *miR-27b-3p* and *miR-539-5p* were downregulated (Figure 8B). *ANGPT1*, *PHGDH, ENTPD3, EPAS1*, and *IL-33* were upregulated (Figure 8C). The results were consistent with those of the microarray analyses, confirming the reliability of the microarray data (Figure 8D).

### MiRNAs were DE during osteogenic differentiation of human ligament fibroblasts

**Figure 9:**
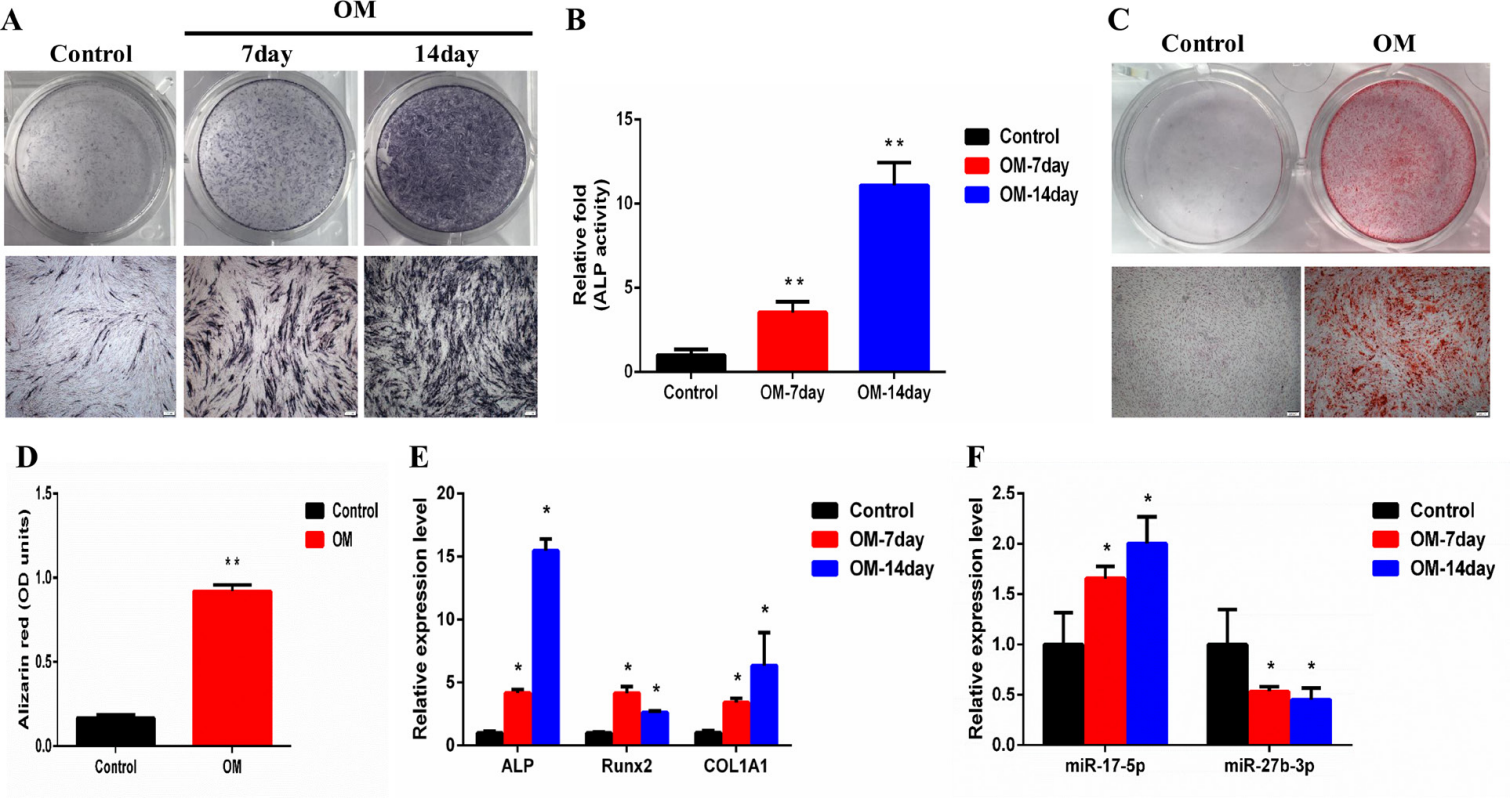
MiR-17-5p and miR-27b-3p were differentially expressed during osteogenic differentiation of human ligament fibroblasts (**A**) ALP in fibroblasts was stained using the BCIP/NBT kit after the cells were incubated in OM for 7 and 14 days. Scale bar = 200 μm. (**B**) The ALP activity of fibroblasts was measured after they were incubated in OM for 7 and 14 days. (**C**) Fibroblasts were incubated in OM for 21 days, and then the mineralized nodules were stained using alizarin red S (ARS). Scale bar = 200 μm. (**D**) Mineralization was quantified by extraction of ARS dye with 10% cetylpyridinium chloride. (**E**) The total RNA was isolated on days 7 and 14. Runx2, ALP, and COL1A1 mRNA levels were determined using qRT-PCR and normalized to GAPDH. (**F**) The total RNA was isolated on days 7 and 14. Levels of *miR-17-5p* and *miR-27b-3p* were determined using qRT-PCR and normalized to U6. ^*^P < 0.05; ^**^P < 0.01 compared with controls. ALP, Alkaline phosphatase; GADPH, glyceraldehyde-3-phosphate dehydrogenase; OM, osteogenic differentiation medium; qRT-PCR, Quantitative real-time polymerase chain reaction.

Human ligament fibroblasts at passage 4 were successfully differentiated into osteoblasts when cultured in osteogenic differentiation medium (OM) for 21 days. The alkaline phosphatase (ALP) activity increased on days 7 and 14 (Figure 9B). A similar result was observed by ALP staining (Figure 9A). Alizarin red S (ARS) staining showed the formation of new calcium nodules on day 21 (Figure 9C). A similar result was observed by quantification of ARS (Figure 9D). Furthermore, the qRT-PCR result showed that *ALP, Runx2*, and *COL1A1* were all significantly upregulated on days 7 and 14 (Figure 9E). Finally, the expression of 6 DE miRNAs was detected during the osteogenic differentiation. The result demonstrated that *miR-17-5p* was upregulated, whereas *miR-27b-3p* was downregulated (Figure 9F).

## DISCUSSION

AS is the major subtype of spondyloarthritis with an unknown etiology. Thus, understanding the molecular mechanisms of AS would be of immense importance. Previous studies have focused mainly on protein-coding genes [32]. Although the functional relevance of several miRNAs in the plasma of AS has been proved, the involvement of lncRNA in AS is yet to be defined. The lncRNAs, including lnc-ZNF354A-1, lnc-LIN54-1, lnc-USP50-2, and lnc-FRG2C-3, may be involved in the abnormal osteogenic differentiation of mesenchymal stem cells from patients with AS [33]. However, a comprehensive analysis of DE profiles of lncRNAs in AS has not yet been reported. In this study, a microarray was applied to explore the expression profiles of lncRNAs, miRNAs, and mRNAs in patients with AS. This was a novel study to examine DE lncRNAs in AS. Furthermore, a ceRNA network was constructed that provided biological views of lncRNA–miRNA–mRNA interactions. These data might provide strong evidence for the in-depth exploration of the pathogenesis of AS.

The microarray results showed 661 DE lncRNAs, 22 miRNAs, and 574 mRNAs in the AS and control samples. Most of the identified lncRNAs have not been functionally characterized, whereas most of the DE mRNAs and miRNAs are well known. Therefore, bioinformatics analyses of these mRNAs and miRNAs were conducted to help better understand the pathogenesis of AS and speculate on the function of DE lncRNAs. GO and pathway analyses showed that the DE mRNAs and target genes of DE miRNAs were related mainly to inflammation, cell activation processes, angiopoiesis, and ossification that are clearly associated with AS pathogenesis. The KEGG pathway analysis revealed that 39 signaling pathways exhibited significant differences in hip joint ligaments of patients with AS. Based on the analysis, a few AS-related pathways were detected, including cytokine–cytokine receptor interaction, ubiquitin-mediated proteolysis, JAK–STAT signaling pathway, TGF-β signaling containing the BMP pathway, PI3K–Akt signaling pathway, Hippo signaling pathway, and HIF-1 signaling pathway. These pathways were involved mainly in immune regulation, intercellular signaling, osteogenic differentiation, and protein synthesis and degradation. Previous studies have demonstrated the involvement of BMP signaling pathway in new bone formation in AS [34]. The PI3K–Akt signaling pathway and its downstream targets were reported to be critical regulators of bone formation and involved in the pathogenesis of AS [35]. The JAK–STAT signaling pathway has also been suggested to regulate Th17 cells in AS [36]. JAK2 polymorphisms have been implicated to be associated with AS [37]. A signal-net analysis was performed on significant GOs and pathways to identify important genes involved in the molecular mechanism of AS. The analysis revealed that *ATM* and *ITGA2* exhibited the highest degrees. *ATM* was reported to promote abnormal proliferation of breast cancer–associated fibroblasts through maintaining intracellular redox homeostasis and activating the PI3K–Akt, MEK–ERK, and Wnt/β-catenin signaling pathways [38]. Moreover, *ITGA2* enhanced joint inflammation and cartilage destruction by activating the MEK–ERK signaling pathway [39]. Thus, the signal-net results suggested that these genes were crucial in the pathogenesis of inflammation and ossification in AS.

The CNC network identified some key genes, including *VASH1* and *SPINK5*, and some lncRNAs, including CSNK1D-AS8, NDRG1-AS6, and CD46-AS9. *VASH1*, which served as inhibitors of angiogenesis. They were downregulated in hip joint ligaments of patients with AS, thereby participating in the pathogenesis of AS. An imbalance between angiogenic inducers and inhibitors seems to be an important factor in the pathogenesis of autoimmune diseases. Studies have suggested that angiogenesis might be implicated in AS pathogenesis and serve as a therapeutic target [40]. *SPINK5* belongs to the protease inhibitor family; it is involved in anti-inflammatory and antimicrobial protection of mucous epithelia [41]. CSNK1D-AS8, as a component of Wnt/β-catenin and Hedgehog signaling pathways [42], may be implicated in the regulation of cell development and differentiation [43]. Previous studies have reported that NDRG1-AS6 and CD46-AS9 might participate in the pathology of inflammation by regulating the NF-κB signaling pathway [44, 45].

In this study, a ceRNA network was constructed based on microarray data. For example, as a ceRNA, lncRNA ENST00000529935 competes for binding to *miR-548d-3p*, thereby affecting the expression of *TAP2*. *TAP2*, participating in antigen processing and presentation, was reported to be associated with AS [46, 47]. Additionally, genetic variations within *STXBP6* might influence the response to TNF-α inhibitors in patients with RA [48]. Understanding these novel RNA cross-talks might provide insights into gene regulatory networks with implications in AS.

Recent studies have revealed that several miRNAs might be involved in AS pathogenesis. However, most of the studies were conducted on the plasma. Tissue miRNAs have been noted not only as key molecules in intracellular regulatory networks for gene expression but also as biomarkers for various pathological conditions [49]. This novel study detected miRNAs in tissues of patients with AS. In the present study, several candidate miRNAs were selected to investigate their functions on the osteogenic differentiation of ligament fibroblasts. The experiments showed that *miR-17-5p* was upregulated, whereas *miR-27b-3p* was downregulated. The results were consistent with the microarray data. Previous studies demonstrated that *miR-17-5p* could regulate osteogenic differentiation by targeting several osteogenic markers, such as BMP2, SMAD5, and SMAD7 [50–52]. A recent study showed that *miR-27b-3p* suppressed osteogenic differentiation of maxillary sinus membrane stem cells by targeting Sp7 [53]. The findings indicated that *miR-17-5p* and *miR-27b-3p* were involved in the osteogenic differentiation of ligament fibroblasts. This study hypothesized that these miRNAs might participate in the pathogenesis of new bone formation in AS.

The present study had some limitations. First, the sample size was small. Studies with a large number of patients with AS are needed for obtaining better results. Moreover, the mechanism by which the two miRNAs regulate the osteogenic differentiation of ligament fibroblasts is unknown. Further studies should address these questions.

In summary, the present study provided comprehensive lncRNA, miRNA, and mRNA profiles for AS hip joint ligaments. Bioinformatics approaches were used to predict the potential functions of DE mRNAs and initially explore their roles in the pathogenesis of AS. The cell experiment indicated that miRNAs might participate in ossification. These findings might provide new insights into the pathogenesis of AS and lay a foundation for further functional research. However, the biological functions and specific molecular mechanisms of DE lncRNAs in the pathogenesis of AS warrant further exploration.

## MATERIALS AND METHODS

### Patients and samples

Hip joint ligament tissue samples were obtained from 20 HLA-B27-positive patients with AS undergoing total hip replacement at the Department of Orthopedics in Changhai hospital between January 2013 and December 2016. All of them fulfilled the modified New York criteria for AS [54]. Additional 34 patients with femoral neck fracture who underwent total hip arthroplasty were used as controls. The characteristics of all the patients are shown in Table 3. The study was approved by the ethics committee of Changhai hospital. Both patients and controls provided written informed consent. All samples were divided into two parts: five pairs of tissues were used for microarray analysis, and the remaining tissues were used for validation by qRT-PCR.

**Table 3:** Characteristics of patients with AS

Characteristics	Total sample (*N* = 20)
Age(year) Sex (M/F) (*n*) BMI (kg/m^2^) Duration of AS (year) ESR (mm/h) CRP (mg/L)	41.3 ± 10.7 19/1 20.5 ± 1.6 11.3 ± 3.2 15.6 ± 11.0 10.1 ± 7.1

AS, Ankylosing spondylitis; BMI, body mass index; CRP, C-reactive protein; ESR, erythrocyte sedimentation rate.

### RNA extraction and microarray assay

The total RNA was extracted from frozen tissues using the RNeasy kit (QIAGEN, Hilden, Germany) according to the manufacturer’s instructions. An ND-1000 spectrophotometer (NanoDrop Technologies, DE, USA) was used to measure the concentration and purity of RNA. RNA integrity was measured using agarose gel electrophoresis. The microarray experiments were performed using the Affymetrix GeneChip Human Transcriptome Array (Affymetrix, MA, USA). The probes on the array covered all known coding transcripts (including alternative splice variants) and more than 40,000 noncoding transcripts (including lncRNAs, miRNAs, and small nucleolar RNAs). The cDNA labeling, microarray analysis, and bioinformatics analysis were performed by Genminix Informatics (Genminix, Shanghai, China). Briefly, the sample labeling, hybridization, and washing were performed based on the user manual. The arrays were scanned using the GeneChip scanner 3000 (Affymetrix). Data were analyzed using the Affymetrix Expression and Transcriptome Console Software. A random variance model *t* test was used to filter the DE transcripts for experimental and control groups in the cases of small samples. After significance analysis and false discovery rate (FDR) analysis, the aberrantly expressed transcripts (lncRNAs, miRNAs, and mRNAs) were identified using *P* values (< 0.05). The DE gene lists are shown in the Supplementary Dataset 1–3. The raw data for the microarray (.CEL) can be downloaded from Baidu SkyDrive (http://pan.baidu.com/s/1pKNKoqf).

### GO and KEGG pathway analyses

The DE mRNAs and the predicted target genes of DE miRNAs were annotated in terms of their GO categories and KEGG pathways using the web-based tool, Database for Annotation, Visualization, and Integrated Discovery (http://david.abcc.ncifcrf.gov/). Fisher’s exact test and *χ*^2^ test were used to select significant GO categories and KEGG pathways, and the threshold of significance was defined by *P* < 0.05 (the FDR was used to correct the *P* value).

### MiRNA target gene prediction and network construction

Target genes of the DE miRNAs were predicted using the combined miRNA target prediction databases including miRanda (http://www.microrna.org/) and TargetScan (http://www.targetscan.org/). Only the target genes that were identified in both databases were considered further to reduce the false positivity. The predicted targets of deregulated miRNAs were compared with those of the deregulated mRNAs from the mRNA expression profile data to detect overlaps. The interactive network of miRNAs and actually deregulated target mRNAs was visualized using the Cytoscape software (http://www.cytoscape.org/). The miRNA–GO network was built according to the relationships between genes and significant GOs and the relationships among miRNAs and GOs.

### Gene signal transduction network and lncRNA–mRNA co-expression network

The gene signal transduction network (signal-net) was constructed based on the data of DE genes. The KEGG database was used to analyze functional gene interactions. In the network, nodes represented genes (protein, compound, and so forth) and edges represented relation types between the nodes (activation or phosphorylation, and so forth). The degree of a gene was defined as the number of directly linked genes within a network, which could evaluate the relative significance of a gene in the network. In the signal-net analysis, the genes were characterized by measuring their “betweenness centrality”: the number of times a node was located in the shortest path between two other nodes. The CNC networks were constructed to identify interactions among genes and lncRNAs. They were built according to the normalized signal intensity of specific expression genes. In the present study, only lncRNA–mRNA pairs with the Pearson correlation coefficient (PCC) value ≥0.98 and *P* <0.05 were retained for network construction and further analysis. In considering different networks, the core regulatory genes were assessed by the degree of difference they showed in their roles in the AS and control networks, which was measured with the variable Diffk (difference in normalized connectivities). The co-expression networks were drawn using the Cytoscape software.

### Construction of the ceRNA co-expression network

The ceRNA network was constructed based on the ceRNA theory as follows: (1) negatively correlated mRNA–miRNA pairs were screened, where the mRNAs were the targets of the miRNAs; (2) negatively correlated lncRNA–miRNA pairs were screened, where the lncRNAs were the targets of the miRNAs; (3) mRNA–lncRNA integrations were evaluated by screening mRNA–lncRNA pairs with positive co-expression relationships using PCC; and (4) both lncRNA and mRNA in an lncRNA–mRNA pair were targeted and negatively correlated with a common miRNA. The lncRNA–miRNA–mRNA network was constructed by assembling all lncRNA–miRNA–mRNA triplets, also known as ceRNAs.

### Cell culture and osteogenic differentiation

Primary fibroblasts (*N* = 3) were isolated from hip ligament tissues using the previously described method with some modifications. The ligaments were cut into 0.3-cm^3^ pieces, washed twice with sterile phosphate-buffered saline (PBS) to remove blood, and centrifuged at 1500 revolutions per minute (rpm) for 5 min. The ligaments were resuspended in fetal bovine serum (FBS; HyClone, GE Healthcare, Buckinghamshire, UK) and then seeded into 25-cm^2^ flasks for 4 h at 37°C. Then, FBS was replaced with Dulbecco’s modified Eagle’s medium (DMEM; HyClone) containing 15% FBS, 100 U/mL penicillin, and 100 µg/mL streptomycin. After cells adhered, the ligament pieces were removed, and the culture medium was replaced every 3 days thereafter. The passages from 3 to 8 were used for all subsequent experiments. Flow cytometry (BD FACSCalibur, NJ, USA) was used for fibroblast identification marked by CD29 and CD90, as described in previous studies (Supplementary Figure 1). For osteogenic differentiation, the fibroblasts were seeded in 24-well plates in a growth medium consisting of DMEM with 10% FBS. When the culture reached a confluence of 80%–90%, the medium was changed to OM containing 100nM dexamethasone, 10 mM β-glycerophosphate, 50 μg/mL of ascorbic acid, and 10% FBS. The fibroblasts were cultured in OM for up to 21 days, and the medium was replaced every 3 days.

### ALP activity and staining

The ALP activity in the fibroblasts was detected using an ALP activity kit (Jiancheng Biotech, Nanjing, China). Briefly, the fibroblasts were lysed in a lysis buffer containing 1% Triton X-100, 20mM Tris–HCl (pH 7.5), and 150mM NaCl. The lysate was centrifuged at 12,000 rpm at 4°C for 5 min, and the supernatant was incubated with reaction buffers at 37°C for 15 min. Then, the coloration solution was added, and the absorbance was measured at 520 nm. The total protein concentration was determined using a bicinchoninic acid protein assay kit (Pierce, MA, USA). The relative ALP activity was expressed as units per gram of protein per 15 min. The ALP staining assay was performed using a BCIP/NBT ALP Color Development Kit (Beyotime, Shanghai, China). The fibroblasts were washed with PBS twice and fixed with 95% ethanol for 15 min. The cells were washed with PBS twice and incubated with ALP dye solution in the dark for 30 min. Then the reaction was stopped using distilled water, and the plate was dried before taking a photo.

### ARS staining and quantification

Fibroblasts were washed with PBS and fixed with 95% ethanol for 15 min. The cells were then stained with 1% ARS (pH 4.3) for 15 min and washed three times with distilled water to remove nonspecific staining. The stained cells were visualized on photographs and observed under a microscope. For ARS quantification, the calcium deposition was destained with 10% cetylpyridinium chloride (Sigma–Aldrich, MO, USA) in 10 mM sodium phosphate for 1 h at room temperature. The absorbance was measured using a microplate reader at 562 nm.

### qRT-PCR

The total RNA was isolated with a TRIzol reagent (Invitrogen) according to the manufacturer’s instructions. For lncRNAs and mRNAs, the total RNA was reverse transcribed into cDNA using a PrimeScript RT reagent kit (Takara, Japan). Ordinary qRT-PCR was performed on a LightCycler 480 PCR system (Roche, Germany) using SYBR Premix Ex Taq II (Takara, Japan). MiRNA qRT-PCR was performed using a Bulge-Loop miRNA qRT-PCR Starter Kit (RiboBio, Guangzhou, China). The lncRNA/mRNA and miRNA PCR results were quantified using the 2^-ΔΔCt^ method, with normalization using glyceraldehyde-3-phosphate dehydrogenase (GAPDH) and U6, respectively. Fold changes were shown as means ± standard deviation (SD) from three independent experiments. The primer sets specific for miRNA and lncRNA were purchased from Ribobio (Guangzhou, China). The forward and reverse primers for some genes are presented in Supplementary Table 1.

### Statistical analysis

The statistical analysis was performed using the SPSS 19.0 software (SPSS Inc., IL, USA). All data were expressed as mean ± SD. The Student *t* test was performed to analyze the microarray and qRT-PCR data. A *P* value < 0.05 was considered statistically significant.

## SUPPLEMENTARY MATERIALS FIGURES AND TABLE









## Abbreviations

AS: Ankylosing spondylitis
lncRNA: long noncoding RNA
miRNA: microRNA
mRNA: messenger RNA
ceRNA: competing endogenous RNA
ncRNA: noncoding RNA
DE: differentially expressed
OA: osteoarthritis
RA: rheumatoid arthritis
GO: Gene Ontology
KEGG: Kyoto Encyclopedia of Genes and Genomes
CNC: coding–noncoding gene co-expression
qRT-PCR: quantitative real-time polymerase chain reaction
OM: osteogenic differentiation medium
PCC: Pearson correlation coefficient
ALP: alkaline phosphatase
ARS: alizarin red S
