# Supplementary material for: Differentially expressed mRNAs, lncRNAs, and miRNAs with associated co-expression and ceRNA networks in ankylosing spondylitis
Source: Oncotarget. 2017 Nov 27;8(69):113543–57. doi: 10.18632/oncotarget.22708 (PMC5768345; doi:10.18632/oncotarget.22708)
Supplement: Supplementary file 1 [file oncotarget-08-113543-s001.pdf]

Differentially expressed mRNAs, lncRNAs, and miRNAs with associated co-expression and ceRNA networks in ankylosing spondylitis

SUPPLEMENTARY MATERIALS

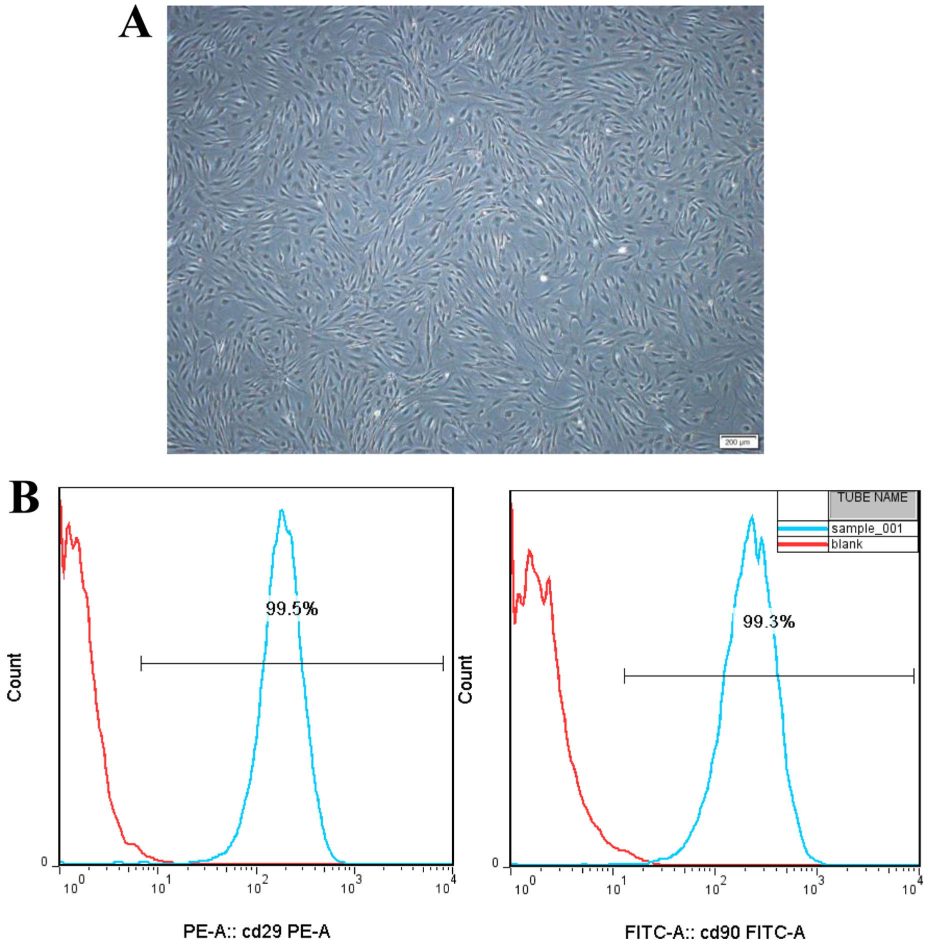

Supplementary Figure 1: Morphology and phenotype of hip ligament–derived fibroblasts. (**A**) Morphologic features of fibroblasts were assessed by microscopy. Scale bar = 200 µm. (**B**) Phenotypes of fibroblasts were determined by flow cytometry for the expression of CD29 and CD90. PE, Phycoerythrin; FITC, fluorescein isothiocyanate.

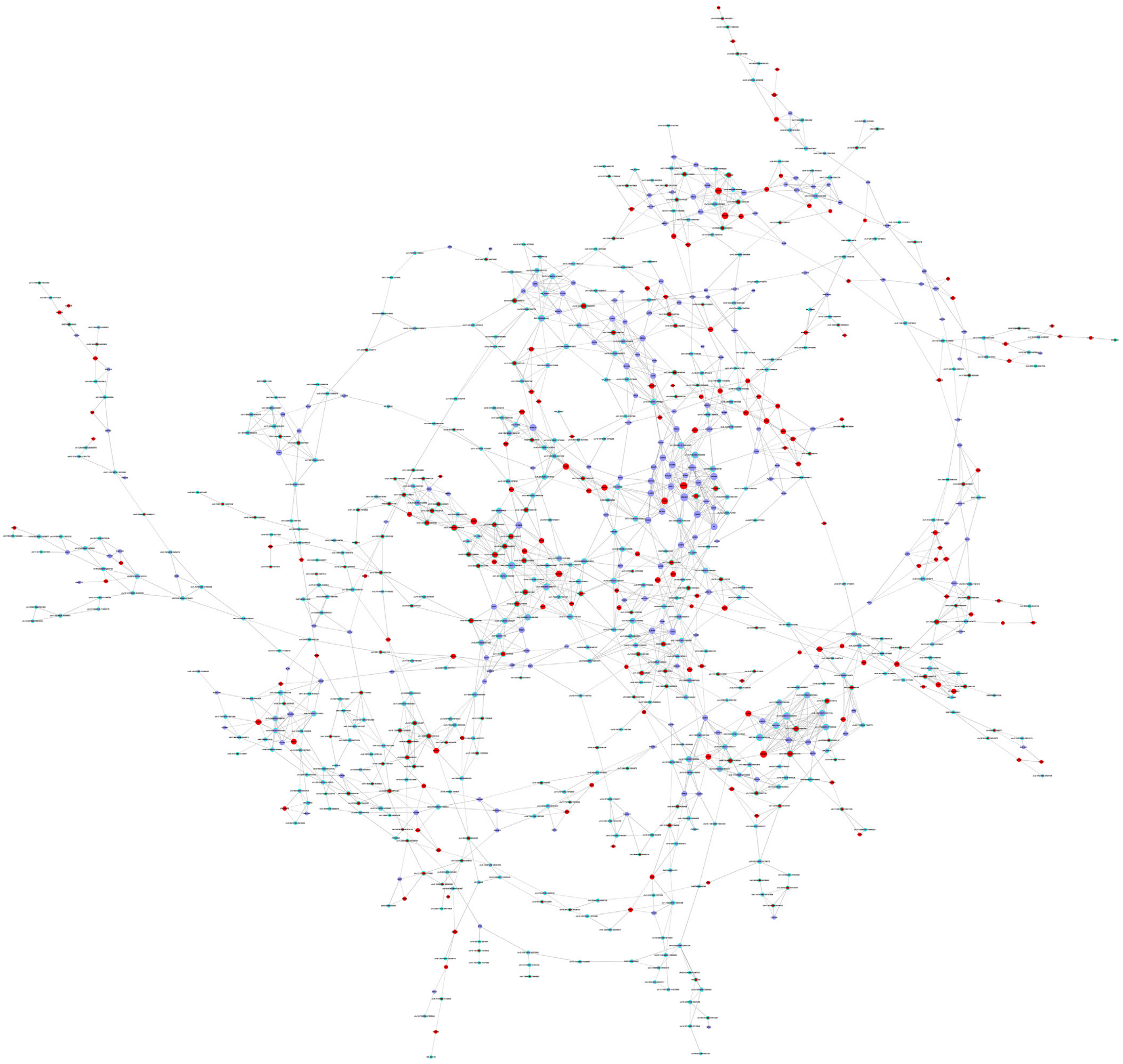

Supplementary Figure 2: Co-expression network of mRNAs and lncRNAs in patients with AS. *Circles* represent upregulated (*red*) and downregulated (*purple*) genes. The lncRNA genes are encircled in green. The lines represent the regulatory relationships between genes (solid lines represent positive correlations, and dotted lines represent negative correlations). The circle size represents the degree of centrality.

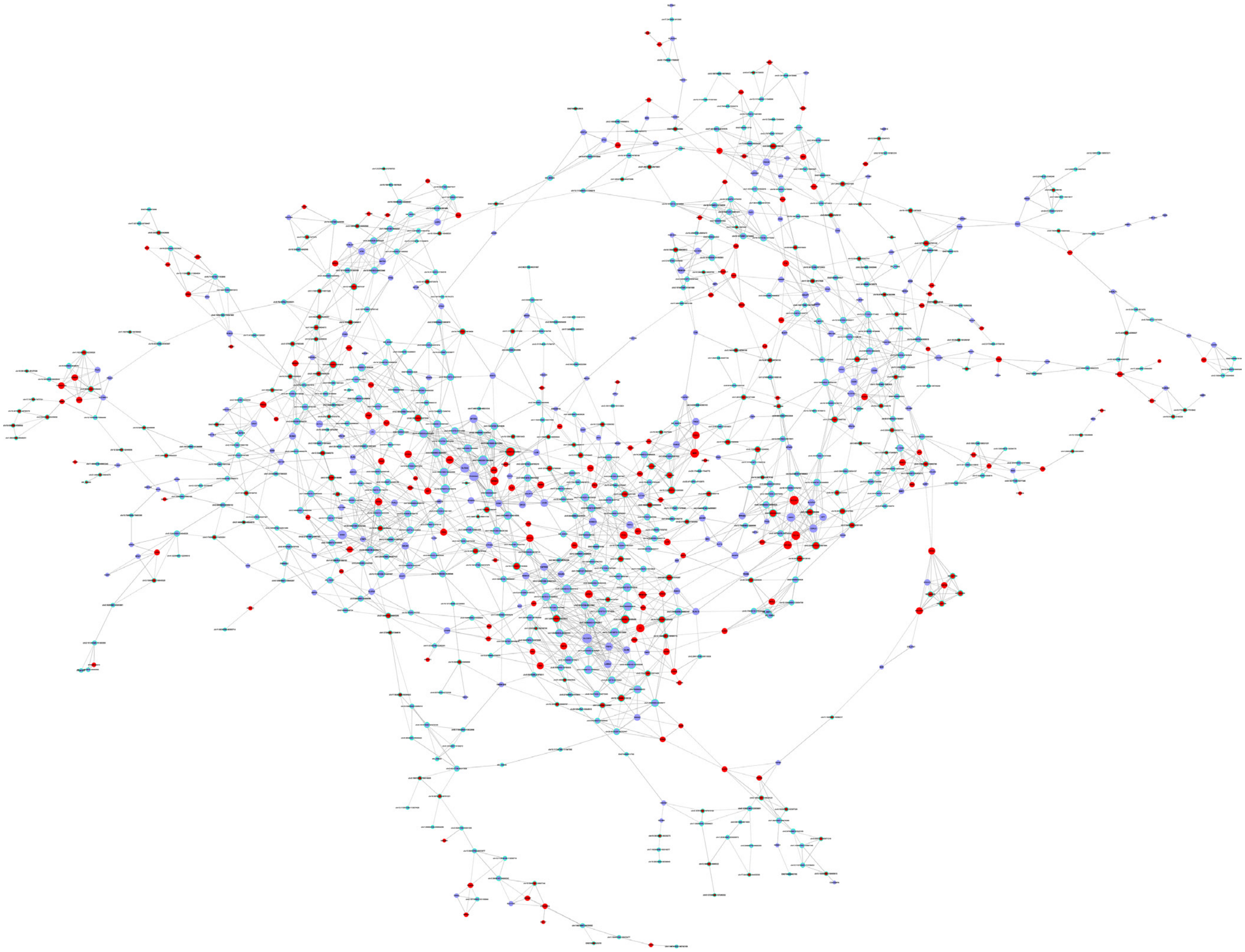

Supplementary Figure 3: Co-expression network of mRNAs and lncRNAs in controls. *Circles* represent upregulated (*red*) and downregulated (*purple*) genes. The lncRNA genes are encircled in green. The lines represent the regulatory relationships between genes (solid lines represent positive correlations, and dotted lines represent negative correlations). The circle size represents the degree of centrality.

Supplementary Table 1: PCR primers used in this study

Gene symbol	Forward	Reverse
GAPDH	CATGAGAAGTATGACAACAGCCT	AGTCCTTCCACGATACCAAAGT
ANGPT1	TTAATAATATGCCAGAACCCAA	CAAAAATAAACTCATTCCCCAG
ENTPD3	AAGAAGTGGGGAATAGCAGCATA	GGGTAGTGAGGTCTCTGAAGAACAA
EPAS1	CGGTCATCTACAACCCTCGC	TTCTCAGACACAGCCCCCTT
IL-33	GTGACGGTGTTGATGGTAAGAT	AGCTCCACAGAGTGTTCCTTG
PHGDH	CACGACAGGCTTGCTGAATGA	CTTCCGTAAACACGTCCAGTG
ALP	AGCCCTTCACTGCCATCCTGTAT	CGCCTGGTAGTTGTTGTGAGCAT
Runx2	CCAGGCAGTTCCCAAGCATTTCA	GGTAGTGAGTGGTGGCGGACATA
COL1A1	GAGACTGGTGAGACCTGCGTGTA	GCCGCCATACTCGAACTGGAATC

Supplementary Dataset 1: Differentially expressed mRNAs. See Supplementary_Dataset_1

Supplementary Dataset 2: Differentially expressed miRNAs. See Supplementary_Dataset_2

Supplementary Dataset 3: Differentially expressed lncRNAs. See Supplementary_Dataset_3
